# Extending beyond Policy: Reaching UNAIDS’ Three “90”s in Malawi

**DOI:** 10.3389/fpubh.2018.00069

**Published:** 2018-03-16

**Authors:** Zengani Chirwa, Florence Kayambo, Lolade Oseni, Marya Plotkin, Cyndi Hiner, Chimwemwe Chitsulo, Kelly Curran, Thokozani Kalua, Stacie C. Stender

**Affiliations:** ^1^Jhpiego Malawi, Lilongwe, Malawi; ^2^Jhpiego, Baltimore, MD, United States; ^3^Johns Hopkins Bloomberg School of Public Health, Baltimore, MD, United States; ^4^Malawi Ministry of Health HIV and AIDS Department, Lilongwe, Malawi

**Keywords:** HIV prevention, policy innovation, reaching the three “90”s, HIV testing, viral load testing, Malawi, sub-Saharan Africa

## Abstract

Malawi, like other countries with a generalized HIV epidemic, is striving to reach the ambitious targets set by UNAIDS known as the three 90’s for testing, provision of antiretroviral therapy and viral suppression. Assisted by Malawi’s progressive policies on HIV/AIDS, it appears possible that Malawi will attain these targets, but only by employing innovative program approaches to service delivery which help fill policy gaps. This article describes how a dedicated cadre of layperson testers and HIV-positive peers appears to have helped attain increases in HIV and viral load testing and retention in care in four districts in Malawi, and situates these innovations in a policy framework analysis.

## Introduction

Malawi, a southern African country of 17.8 million people (1), has an adult HIV prevalence of 10.6% (2) and an estimated 980,000 people living with HIV (PLHIV) (3). Like other countries with a generalized HIV epidemic, Malawi is striving to reach the ambitious target known as “90-90-90” by 2020, whereby 90% of PLHIV know their status, 90% of PLHIV with known HIV status receive sustained antiretroviral therapy (ART), and 90% of people receiving ART achieve viral suppression (4). It is possible that Malawi will attain this goal in the timeframe. Currently, roughly 73% of Malawians living with HIV know their status, 89% of Malawian PLHIV are on ART, and 91% of these are virally suppressed (2).

Malawi is recognized for adopting and implementing progressive HIV policies, being the first country in sub-Saharan Africa to roll out a WHO-recommended “public health approach” to HIV prevention (5), detailed in Malawi’s National HIV and AIDS Strategic Plan (2011–2016) (6). This progressive policy approach has led to positive changes. Introduction of ART, which began in 2004, led to declines in mortality: in Karonga district, there was a 25% decline in the proportion of deaths attributable to HIV/AIDS in the 2 years following introduction of ART (7, 8), and all-cause adult mortality fell by 35% (9). More effective means of prevention of mother-to-child-transmission of HIV through provision of ART to all HIV+ pregnant women—known as Option B+—was conceived and implemented first in Malawi. ART coverage among HIV+ pregnant women increased from 49 to 85% following introduction of Option B+ in 2011–2014 (10).

In Malawi, there is high agreement between national and WHO policies for HIV testing and retention in care. However, there are areas of disconnect in the implementation of these policies, which are likely to contribute to attrition across the HIV diagnosis, care, and treatment continuum. For example, there is a lack of clear guidelines on how laypeople can be involved in linking people to care and treatment. The policy on home visits for clients who miss appointments is also unclear, reducing opportunities to return clients to care. In light of the ambitious 90-90-90 targets, these gaps in policy-to-practice sometimes necessitate implementation approaches which extend beyond what is written in policy guidance.

The aim of this article is to compare a global policy framework with findings from four districts in Malawi to inform discussion about how Malawi and other sub-Saharan African countries can reach the 90-90-90 targets. We present examples from a community to facility HIV prevention, care, and treatment program in light of WHO and Malawi recommendations, discuss challenges, and highlight actionable recommendations for program innovations which build upon policy to effectively link those in need of services to care.

## Policy and Program Application

### Program Setting and Description

**Figure 1:**
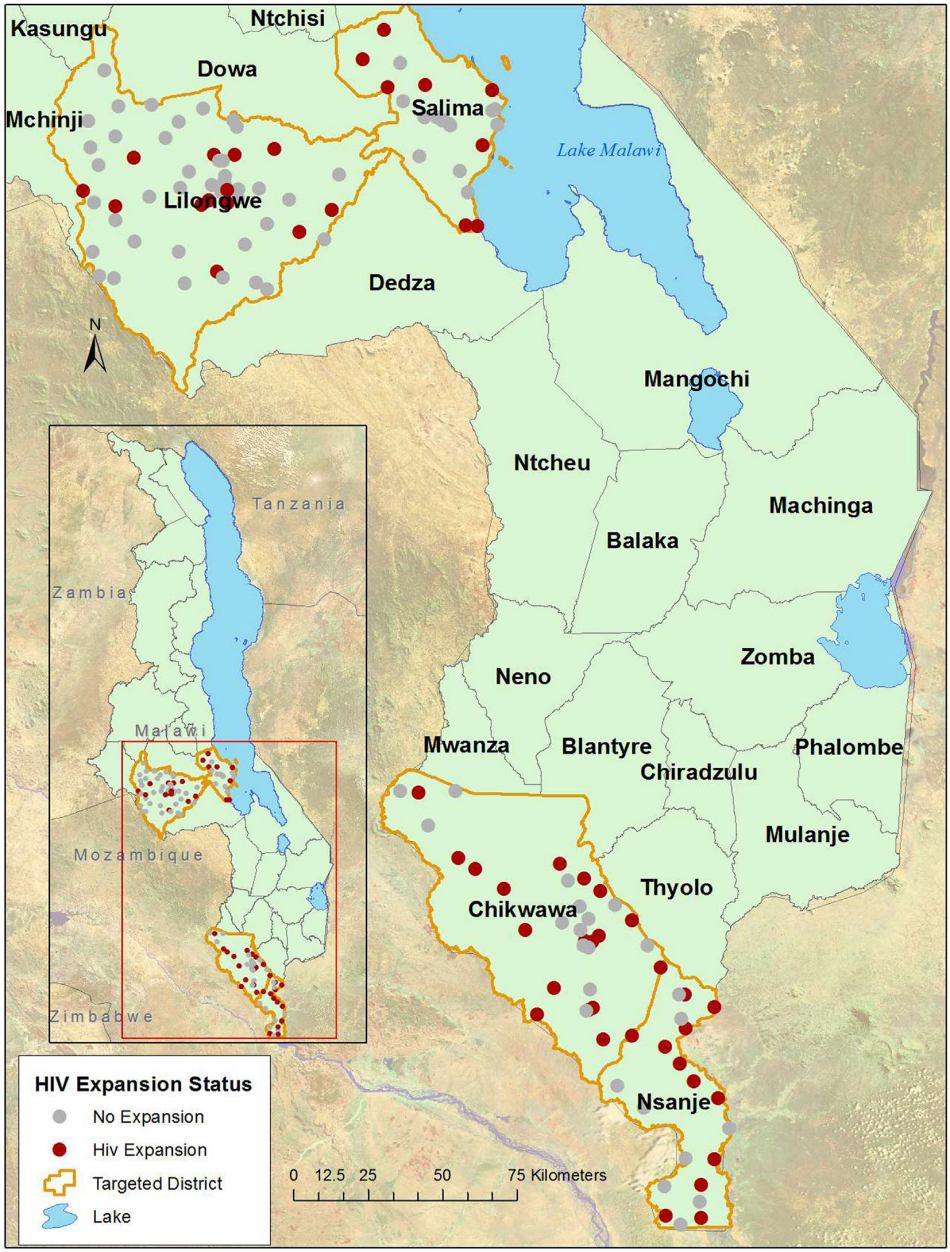
Map of SSDI program districts and facilities.

The Support for Service Delivery-Integration-Services HIV Expansion Project (SSDI-HIV) was funded by the United States Government through the Presidents Emergency Plan for AIDS Relief, administered by USAID and implemented by Jhpiego. SSDI-HIV focused on expanding access to HIV and tuberculosis testing, care, and treatment in 54 health facilities in four districts in Malawi (Chikwawa, Lilongwe, Nsanje, and Salima) from April 2015 to September 2016 (Figure 1). These facilities constitute 42% of facilities in the four districts, including all facilities in Chikwawa and Nsanje and roughly one quarter in Lilongwe and Salima. The program built capacity of district and facility-level Ministry of Health staff through in-service training, clinical mentorship, supportive supervision, infrastructure improvements, and peer support services to PLHIV.

### Toward the First ’90: Establishment of a New Cadre of HIV Testers

In 2015, to increase HIV testing access, the Malawi MOH suggested establishment of a cadre called HIV diagnostic assistants (HDAs), non-health-care providers employed to provide facility-based HIV testing services (HTS). HDAs were to have a minimum secondary school education. They received a 4-week training. The MOH intended that one or two HDAs would be placed at each facility, to ensure a minimum of two counselors. HDAs were responsible for facility-based HTS, collection of dried blood spot specimens for viral load testing and early infant diagnosis of HIV-exposed infants, and contributed to quality control for mobile outreach testing. In 2016, SSDI-HIV recruited, trained, and deployed 106 HDAs to 54 health facilities, with larger facilities having up to four.

### Toward the Second ’90: Establishing a Group of Expert Client Volunteers

In 2015, SSDI-HIV introduced a new strategy to strengthen retention in care and re-engage those lost to follow-up by establishing a volunteer cadre called Expert Clients. Clients who were HIV+ and on ART for at least 2 years were recruited to support community outreach. Unlike HDAs, establishment of the Expert Client volunteer group was not requested by the government of Malawi and was not considered a cadre in the formal health system, but their work was recognized and facilitated by health facility staff. From June to December 2015, 619 Expert Clients were recruited, trained, and deployed in communities surrounding facilities. They conducted outreach with a primary objective of reaching loss to follow up (LTFU) clients, who were contacted by phone and/or visited at home, provided with tailored counseling, and physically escorted to the facility should they choose. Originally, Expert Clients were not paid, but in 2016 they started receiving roughly $70 per month.

### Toward the Third ’90: Increasing Viral Load Testing through Expert Clients and “Catch Up” Testing

Viral load testing services were bolstered by referrals by Expert Clients, within-facility referral by HDAs and expanded testing services in project-supported health facilities. Facilities broadened the Government of Malawi’s viral load testing schedule, the “milestone approach.” This schedule, started in 2011, calls for viral load testing for all clients on ART after 6 months on treatment, again at 2 years, and every 2 years thereafter. This modification of WHO’s annual viral load testing recommendation was due to insufficient numbers of trained lab technicians and cost. SSDI conducted “catch up” viral load testing for all clients on ART for 6 months or more who did not have documented viral load test result.

## Methods

### Policy Analysis

Church et al. (11) introduce a policy review conceptual framework specific to HIV services which breaks down factors affecting policy and practice in the context of national policy environment. These factors in turn affect the continuum of care from diagnosis to retention on treatment. Dasgupta et al. (5) analyze policy compliance, using this policy framework, among six facilities in northern Malawi.

**Figure 2:**
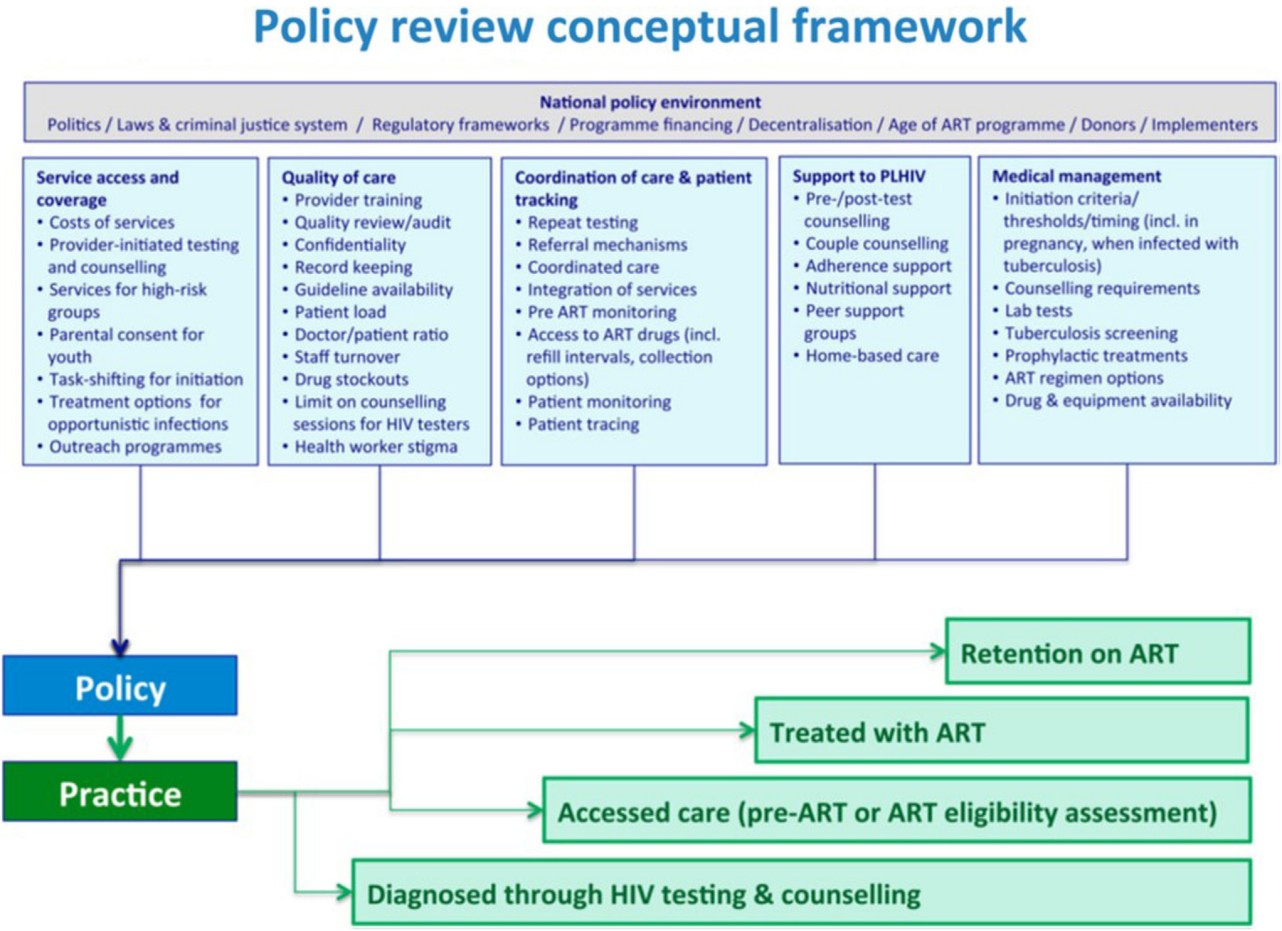
Policy review conceptual framework, adapted from Church et al. (11).

Church et al.’s conceptual framework (Figure 2) forms the basis for our comparison of policy and “practice extending policy” for populations served by SSDI-HIV. Based on the three 90s, we examined utilization of services from the pre- and post-program implementation periods. We then use the policy review framework to discuss the strengths and gaps of specific program approaches.

### Data Sources

The data come from SSDI-HIV program reports and the health management information system (HMIS) database maintained by the HIV and AIDS Department, Malawi MOH and are used with permission of the MOH. The data presented are counts of clients attending services, combined across the 54 program health facilities. To monitor service delivery in SSDI-HIV supported health facilities, we used standardized, national MOH tools and selected program tools. We compared service delivery data for the 15-month project period (July 2015–September 2016) with the preceding 15 months in the same facilities.

## Policy Implications

### Comparison of Policy to Service Delivery in Program Area

Table 1 presents a comparison of WHO guidance, Malawi policy guidance, findings from the policy adherence paper by Dasgupta et al., and practice during the implementation of the SSDI-HIV program, presented using the UNAIDS 90-90-90 targets.

**Table 1:** Comparison of selected WHO, Malawi policy guidance, and practice introduced in the SSDI-HIV program.

WHO guideline	Malawi policy guidance	Compliance with policy [described in Dasgupta et al. (5)]	Practice beyond the policy in facilities during SSDI-HIV (2014–2016)
**90% of people living with HIV (PLHIV) know their status—first 90**			

Provider-initiated testing and counseling is standard for all clients, including at antenatal clinic (ANC) (*Guidance on provider initiated HIV testing and counseling in health facilities, WHO, 2007*)	“Health providers are asked to ascertain HIV status for all clients attending health services (provider-initiated testing and counseling). Clients in ANCs are especially encouraged for HIV testing, due to the Option B+ policy” (*Clinical Management of HIV in Children and Adults: Malawi Integrated Guidelines for Providing HIV Services in ANC, Maternity, Under 5, FP, Exposed Infant/Pre-ART, ART Clinics*; MOH Malawi, 2011) (12)	Partial (all facilities with ANC HIV testing. Most facilities offer provider-initiated testing, and some facilities offer on an “opt-in” basis)	Dedicated HIV diagnostic assistants (HDAs) providing expanded access to HIV testing in facility setting; Expert Clients providing escorted and unescorted referrals from community to facility for HIV testing services (HTS)

In a generalized epidemic, WHO recommends community-based HTS with linkage to prevention, treatment, and care services in addition to routine provider-initiated testing and counseling for all populations, and particularly for key populations (*Consolidated Guidelines on the Use of Antiretroviral Drugs for Treating and Preventing HIV Infection, 2nd edition, WHO, 2016*)	“Community-based HTS can be conducted in various modes including campaign, home based, door to door, workplace, mobile, outreach, and school/educational institutions. Strategic and targeted services recommended to ensure high yield” (*Malawi HIV Testing Services Guidelines, Malawi MOH, 2016*)	Not described in Dasgupta et al.	Expert Clients referred from community to facility setting for testing. HDAs and Expert Clients conducted mobile and outreach community-based HIV testing in markets and with sugar estate workers

**90% of PLHIV are on antiretroviral therapy (ART)—second 90**			

Home visits and other community outreach are recommended as possible within system and human resource constraints. “Bidirectional referral is essential so that people in stable condition can be moved out of the clinic into the community and those who experience health problems can be referred back to facility care.” (*Consolidated Guidelines on HIV Testing Services, WHO, 2015*)	No policy reference to home visits. “…conduct follow-up group counseling and individual counseling if any sign of poor adherence. Give practical advice: (a) build ARVs into daily routine, (b) ask family or friends for reminders, (c) set a daily alarm on cell phones, and (d) keep a “drug diary” and mark every tablet taken.” (*Clinical Management of HIV in Children and Adults: Malawi Integrated Guidelines for Providing HIV Services in ANC, Maternity, Under 5, FP, Exposed Infant/Pre-ART, ART Clinics*; *MOH Malawi, 2011*)	Complies with minimal version (counseling and practical reminders but no home visits)	Expert Clients trace clients LTFU by phone or through home visits, provide individualized counseling and escort or refer LTFU clients back to care and treatment services

**90% of PLHIV on ART have undetectable viral load—third 90**			

Clients on ART receive a viral load test at 6 months on ART and every year thereafter. (*Consolidated Guidelines on the Use of Antiretroviral Drugs for Treating and Preventing HIV Infection, 2^nd^ edition, WHO, 2016*)	Clients have viral load testing done at 6 months after starting ART, after 2 years on ART, and every 2 years thereafter. (*Clinical Management of HIV in Children and Adults: Malawi Integrated Guidelines for Providing HIV Services in ANC, Maternity, Under 5, FP, Exposed Infant/Pre-ART, ART Clinics*; *MOH Malawi, 2011*)	Not described in Dasgupta et al.	Project-supported health facilities implemented “catch up” VL testing (all clients on ART for at least 6 months tested if no test is recorded in their record)

WHO guidelines on provider-initiated testing and counseling assume that HIV testing will be offered to all clients at risk for HIV infection; Malawi policy is even more explicit on this. Dasgupta et al. noted that adherence to standards for testing was partial in the six facilities assessed: HIV testing was universally offered in ANC, but not in every department. Some facilities offered HIV testing on an “opt-out” rather than “opt-in” basis, whereby individuals are tested unless they decline the test, rather than tested when they request the test. The policy guidance, together with the gap described by Dasgupta et al., was the foundation for the Ministry of Health’s decision to recommend dedicated HDAs in program facilities.

WHO guidelines (2016) recommend home visits following signs of poor adherence among ART clients. While Malawi policy guidance (2011) does not specifically mention home visits, it describes group or individual counseling for clients with poor adherence and gives suggestions for improving adherence to treatment. In the policy analysis by Dasgupta et al., facilities complied with the minimal version of national policy but did not conduct home visits. Using Expert Clients to conduct follow-up and outreach was an attempt to bridge that gap.

WHO guidelines (2016) recommend annual routine viral load testing, with the first test done after 6 months on ART and then every year after that. The Malawi 2011 and 2014 guidelines recommend a first test at 6 months on ART, a second test at 2 years on ART, and testing repeated every 2 years thereafter. In practice, this meant that clients could be on ART for 2–3 years without receiving a viral load test if they did not have their initial test after 6 months on ART. Many facilities found that clients on ART more than 6 months were not being tested since their first milestone—as described in the guidelines—had passed. In response, the SSDI-HIV program introduced “catch up” viral load testing to all clients on ART for more than 6 months. Catch up testing was linked with home visits by the Expert Clients.

### Service Delivery in Program Districts

The intent of this section is to examine service delivery data from before and during program implementation to identify differences (allowing for the non-randomized nature of this assessment, the changes may not be completely due to the program). We compared service statistics in the 15 months before implementation of SSDI-HIV with those after 15 months of implementation (Table 2).

**Table 2:** HIV testing, enrollment and retention in care and treatment, and viral load testing before and during SSDI program implementation.

	15 months pre-program intervention (April 2014–June 2015)	15 months of program intervention (July 2015–September 2016)
**HIV testing**		
Overall number of people tested for HIV in the four districts	232,449	305,115
Number of clients tested for HIV by HIV diagnostic assistants (HDAs)	Not applicable (no HDAs)	183,589
HIV prevalence among those tested	7.9%	7.8%

**Antiretroviral therapy (ART) uptake and retention**		
People initiated on ART	12,061	13,963
Loss to follow-up individuals identified by Expert Clients	0	8,929
Loss to follow-up clients brought back to care by Expert Clients	Not applicable (no Expert Clients)	6,187

**Viral load testing**		
Viral load tests conducted	38,496	55,421
Viral load tests conducted by HDAs	Not applicable (no HDAs)	18,149

Based on HMIS data from 2015, the total catchment population in SSDI-supported facilities was 1,282,652. Although no official figures on the number of estimated PLHIV in the four districts was available, by applying zonal prevalence figures derived from Malawi Ministry of Health (2), we estimate that there were 144,233 HIV-infected persons living in these districts in 2015.

There was a 31.3% increase in HIV testing uptake during implementation, compared with the period preceding the project. HDAs conducted almost 184,000 HIV tests during the 15-month period, accounting for 60.2% of all HIV tests conducted during the program period in the four districts. 5,400 clients were diagnosed with HIV by the HDAs. The percentage of clients testing positive remained the same.

There was a moderate increase (15.8%) in clients initiated on ART during program implementation. A substantial number of people who had previously initiated ART were traced and contacted: of the 8,929 people identified as lost to follow up, 6,187 (69.3%) were re-engaged in care and restarted on treatment.

Nearly twice as many PLHIV had a viral load test during the SSDI-HIV implementation period compared with the 15 months prior to the program, representing a 44% increase in viral load testing in the four districts. Of the 55,421 people who had a viral load test during the program implementation period, 18,149 people (32.7%) had these conducted by HDAs.

## Discussion

Malawi is on track to meet the UNAIDS 90-90-90 targets, and progressive policy has played an important role. The SSDI-HIV program contributed to increased coverage of services by applying innovative program approaches, some of which were designed by the MOH and some by the program. These innovative approaches adhered to national and/or international guidelines, but also went beyond the letter of the guideline to “practice beyond policy.” The establishment of HDAs as a paid layperson cadre offering HTS coincided with an increase in the number of people tested for HIV in the project districts, which in turn led to a larger number of clients starting ART. While we cannot be certain that the deployment of the HDAs directly caused this increase, it is highly likely that they contributed to the increased testing uptake and initiation on ART in these four districts.

One strength of this approach was that it was jointly implemented with the MOH, facilitating successful integration of the HDAs into the health system. This bodes well for its potential scale up and sustainability.

This approach was not without challenges. The fact that approximately 60% of tests conducted in the program districts were conducted by HDAs suggests that HIV testing tasks were shifted to the HDAs rather than augmenting the MOH counselors’ HIV testing efforts, as was the intention of the MOH. Given a severe health worker shortage in Malawi (13, 14) there may have been displacement, with the number of tests conducted by MOH providers decreasing as ministry staff focused on other tasks. Implementers should be aware of this potential impact for settings with health-care provider shortages—one suggestion is that evaluative criteria may be broadened to include person-hours of health-care provider staff freed up for other clinical duties.

There are limitations in the figures presented. This assessment of available HMIS and program data is not statistically rigorous, as it did not employ a randomized pre- and post-test design. The increase in service delivery seen in the program-supported districts is unlikely to be due solely to the SSDI-HIV program, since there were other implementing partners working in the districts, particularly for viral load testing, which was a major focus of expansion nationally during the program period. Despite the limitations, we feel confident that intensive program implemented in these four districts did contribute to the increase in service delivery coverage. Finally, it is noted that while community volunteers like the Expert Clients have been widely utilized in sub-Saharan African settings including Malawi, sustainability may hinge on learning lessons from past community health worker programs, specifically about remuneration and supervision of community health workers (15).

The HIV testing innovation of dedicated laypeople for testing may have helped move the bar on testing, but did not increase the proportion of people tested who were positive, resulting in a relatively low “yield” of previously undiagnosed HIV-infected individuals both before and during program implementation. On average, 8% of those tested in the four districts were HIV positive, slightly below the national prevalence of 10.6% (2). Although the increase in absolute number of people tested led to an increased number of PLHIV diagnosed and linked to care, the employment of HDAs does not appear to address the need to implement efficient, “high-yield” testing approaches.

The use of Expert Clients for tracing clients LTFU was a program innovation which appeared to be effective, re-engaging in care over 6,000 people who would have otherwise remained lost. This is unsurprising. Successful experiences using peers for adherence to ART, outreach and follow up, and other aspects of HIV-related care have been described in Uganda, Ethiopia (16), and, combined with phone reminders, Malaysia (17). However, challenges to this approach have been described, which mainly stem from the role and function of Expert Clients not being clearly defined within the health system; typically negligible financial and organizational support is offered to these peers (16). The case for sustainability of using Expert Clients for tracing LTFU clients would be stronger if the Government of Malawi would commit to creating a cadre, even a volunteer cadre, and further defining the role of peer counselors in HIV prevention, care, and treatment.

## Actionable Recommendations

Policy guidance is a key foundation, but does not always provide a roadmap of what can and should be done to help countries achieve the UNAIDS 90-90-90 targets. Even Malawi, which has been recognized as a leader in HIV policy, has notable gaps in policy documents. The policy analysis presented here, combined with service delivery information, led us to the following recommendations about the intersection of program innovation and policy:

- Applying policy frameworks such as Church et al. can be an important tool to assess policy and practice gaps. These gaps need to be filled with innovative program approaches if the 90-90-90 targets are to be met.
- Ministries should consider including new cadres in their diagnosis-to-care continuum to meet the 90-90-90 targets. Options include laypeople for testing and expert clients for community-based outreach related to tracing clients lost to follow-up clients and encouraging viral load tests.
- Expert Clients appeared to contribute to the second and third 90s, while the approach of paid laypeople integrated into the health system seemed to work well in contributing to the first 90.
- While in the best possible world, these approaches would be rigorously evaluated, the timeframe of achieving the 90-90-90 targets is very short and in some cases, indicative results must be employed. However, a more rigorous evaluation of these program innovations should be conducted, with a focus on cost effectiveness.

## Conclusion

WHO guidance and Malawian national policy provide clear guidance on what to do, but how to implement such guidance requires reaching beyond written policy to find innovations or best practices that confer the greatest good for the population. Practice beyond policy in these four districts comprised deployment of a paid layperson cadre (HDAs), who assisted with HIV testing and linkage to care in health facilities. The collaboration between MOH and implementing partners in this innovation increased its potential scale and sustainability. This innovation appears to have contributed to increased HIV testing uptake, but may have reduced the time dedicated to HIV testing among existing MOH counselors. Expert Clients were effective in bringing LTFU clients back to care and increasing access to viral load testing. However, the sustainability of Expert Clients may be hampered by a lack of role definition amongst peer counselors. For Malawi, the policy implications mean building on the foundation of progressive HIV policy by solidifying the cadre of dedicated HIV testers, strengthening human resources for health, and adding policy statements to incorporate peer volunteers for HIV support services.

## Author Contributions

This article was drafted by ZC, FK, LO, and MP. Data and maps were compiled by LO and CC. Critical review and revisions were conducted by KC, CH, TK, and SS.

## Conflict of Interest Statement

The authors declare that the research was conducted in the absence of any commercial or financial relationships that could be construed as a potential conflict of interest. The reviewer KC and the handling editor declared their shared affiliation and the review was done completely independently of the editor.

## References

[1] United Nations Statistics Division. Country Profile: Malawi. UNdata Fact Sheet. (2017). Available from: http://data.un.org/CountryProfile.aspx?crName=malawi

[2] Malawi Ministry of Health. Malawi Population-Based HIV Impact Assessment 2015-16. (2016). Available from: http://phia.icap.columbia.edu/wp-content/uploads/2016/09/MALAWI-Factsheet.FIN_.pdf

[3] UNAIDS. HIV and AIDS Estimates: Malawi. (2015). Available from: http://www.unaids.org/en/regionscountries/countries/malawi

[4] UNAIDS. 90-90-90 An Ambitious Treatment Target to Help End the AIDS Epidemic. (2014). 40 p. Available from: http://www.unaids.org/sites/default/files/media_asset/90-90-90_en_0.pdf

[5] Dasgupta AN Wringe A Crampin AC Chisambo C Koole O Makombe S HIV policy and implementation: a national policy review and an implementation case study of a rural area of northern Malawi. AIDS Care (2016) 28(9):1097–109. 10.1080/09540121.2016.1168913 27098107 PMC4950451

[6] Ministry of Health Malawi. Malawi National HIV and AIDS Strategic Plan 2011-2016. Lilongwe (2012).

[7] Jahn A Floyd S Crampin AC Mwaungulu F Mvula H Munthali F Population-level effect of HIV on adult mortality and early evidence of reversal after introduction of antiretroviral therapy in Malawi. Lancet (2008) 371(9624):1603–11. 10.1016/S0140-6736(08)60693-5 18468544 PMC2387197

[8] Floyd S Molesworth A Dube A Banda E Jahn A Mwafulirwa C Population-level reduction in adult mortality after extension of free anti-retroviral therapy provision into rural areas in northern Malawi. PLoS One (2010) 5(10):e13499. 10.1371/journal.pone.0013499 20976068 PMC2957442

[9] Chihana M Floyd S Molesworth A Crampin AC Kayuni N Price A Adult mortality and probable cause of death in rural northern Malawi in the era of HIV treatment. Trop Med Int Health (2012) 17(8):e74–83. 10.1111/j.1365-3156.2012.02929.x 22943382 PMC3443368

[10] Haas AD Tenthani L Msukwa MT Tal K Jahn A Gadabu OJ Retention in care during the first 3 years of antiretroviral therapy for women in Malawi’s option B+ programme: an observational cohort study. Lancet HIV (2016) 3(4):e175–82. 10.1016/S2352-3018(16)00008-4 27036993 PMC4904064

[11] Church K Kiweewa F Dasgupta A Mwangome M Mpandaguta E Gómez-Olivé FX A comparative analysis of national HIV policies in six African countries with generalised epidemics: influences on access to testing, access to treatment and retention in care. Bull World Health Organ (2015) 93(7):457–67. 10.2471/BLT.14.147215 26170503 PMC4490813

[12] Ministry of Health Malawi. Clinical Management of HIV in Children and Adults: Malawi Integrated Guidelines for Providing HIV Services in ANC, Maternity, Under 5, FP, Exposed Infant/Pre-ART, ART Clinics. Lilongwe (2011).

[13] Chimwaza W Chipeta E Ngwira A Kamwendo F Taulo F Bradley S What makes staff consider leaving the health service in Malawi? Hum Resour Health (2014) 12(1):1–9. 10.1186/1478-4491-12-17 24641840 PMC3974437

[14] WHO. African Health Observatory, Regional Office for Africa. Malawi: Fact Sheets of Health Statistics (2016). Available from: http://www.aho.afro.who.int/profiles_information/images/d/d8/Malawi-Statistical_Factsheet.pdf

[15] Hermann K Van Damme W Pariyo GW Schouten E Assefa Y Cirera A Community health workers for ART in sub-Saharan Africa: learning from experience – capitalizing on new opportunities. Hum Resour Health (2009) 7:1–11. 10.1186/1478-4491-7-31 19358701 PMC2672918

[16] Gusdal AK Obua C Andualem T Wahlstrom R Chalker J Fochsen G Peer counselors’ role in supporting patients’ adherence to ART in Ethiopia and Uganda. AIDS Care (2011) 23(6):657–62. 10.1080/09540121.2010.532531 21347887

[17] Abdulrahman SA Rampal L Ibrahim F Radhakrishnan AP Shahar HK Othman N. Mobile phone reminders and peer counseling improve adherence and treatment outcomes of patients on ART in Malaysia: a randomized clinical trial. PLoS One (2017) 12(5):e0177698. 10.1371/journal.pone.0177698 28520768 PMC5433794

